# HGM-4: A new multi-cameras dataset for hand gesture recognition

**DOI:** 10.1016/j.dib.2020.105676

**Published:** 2020-05-08

**Authors:** V.T. Hoang

**Affiliations:** Ho Chi Minh City Open University, Vietnam

**Keywords:** Hand gesture recognition, Image classification, Biometric recognition, Sign language, One hand gesture, Multiple cameras

## Abstract

Gesture recognition technology is rapidly growing in the recent years due to the demands of many application such as computer game and sport, human robot interaction, assistant systems, sign language interpretation and e-commerce. One of the most important of gesture recognition is hand-gesture recognition. For example, it can be used to control all devices (television, radio, air-condition, and doors) by just hand gestures for smart home application. The HGM-4 dataset is built for hand gesture recognition (the full dataset is available from: https://data.mendeley.com/datasets/jzy8zngkbg/4) which contains total 4,160 color images (1280 × 700 pixels) of 26 hand gestures captured by four cameras at different position. The training and testing set are defined to create a benchmark framework for comparing the experimental results.

Specifications table

**Table:** 

Subject	Computer Vision, Pattern Recognition, Artificial Intelligence
Specific subject area	hand-gesture recognition, image classification, biometric recognition, sign language
Type of data	Image (1280 × 700 pixels) in RGB color space
How data were acquired	This dataset contains images that were taken by 4 cameras at different positions by laptop camera, indoor condition.
Data format	RAW
Parameters for data collection	Hand-gesture images are removed background semi-automatically.
Description of data collection	This dataset consists of 4,160 images of 26 gestures acquired by 4 different cameras.
Data source location	Ho Chi Minh City Open University, Ho Chi Minh City, Vietnam
Data accessibility	Mendeley Data https://data.mendeley.com/datasets/jzy8zngkbg/4 http://dx.doi.org/10.17632/jzy8zngkbg.4

## Value of the data

• This dataset is constructed for hand-gesture recognition which contains 26 different gestures corresponding to 26 letters of sign language.
• This is the first dataset containing 4 cameras images for hand-gesture in contrast with the rest pubic datasets.
• Hand gesture recognition might be used from this dataset in supervised and semi-supervised learning context.
• This dataset can be applied to study the hand-gesture recognition problems under multiple views. The potential applications can be used for sign language interpretation, contactless device control.
• We propose three strategies of experimental protocol with one, two and three training sets per gesture. The image from 4 cameras were combined as (training, testing) couples with all possible combination. For example, all the images captured from 1 camera are used for testing while the images from the remaining 3 cameras are used as a training set as the first strategy. This decomposition makes HGM-4 as a first benchmark dataset for multiple cameras hand-gesture recognition.

## 1 Data

Gesture recognition allows to interpret an image or sequence of images, i.e., video into a meaningful description. Among them, hand gesture recognition is the active research topics in machine vision and human robot interaction and has a wide range of potential applications such as video games, medical systems, wearable devices, and multimedia systems [12]. Many different approaches exist based on image analysis can be found in literature. Chansri and Srinonchat [1] present hand gesture of Thai sign language under a complex background using fusion of depth and color video. Maqueda et al. [6] a robust vision-based hand-gesture recognition system using volumetric spatiograms of local binary patterns. Dinh et al. [2] presents hand gesture interface for appliances control in smart home environments based on synthetic hand depth and random forests classifier. Dominioet al. [3] extract and divide the acquired hand images into palm and finger regions. Then, four different image descriptors are extracted and an SVM classifier is associated to recognize the performed gestures. Guan et al. [4] introduce a method by fusing information from multiple cameras to provide reliable hand pose estimation. Just and Marcel, [5] present a comparative study of hand gesture recognition in an isolated, complex, dynamic environment based on Hidden Markov Model. Tavakoli et al. [13] introduce a method to classify hand gestures on wearable devices that use EMG sensors as an input source.

**Fig. 1:**
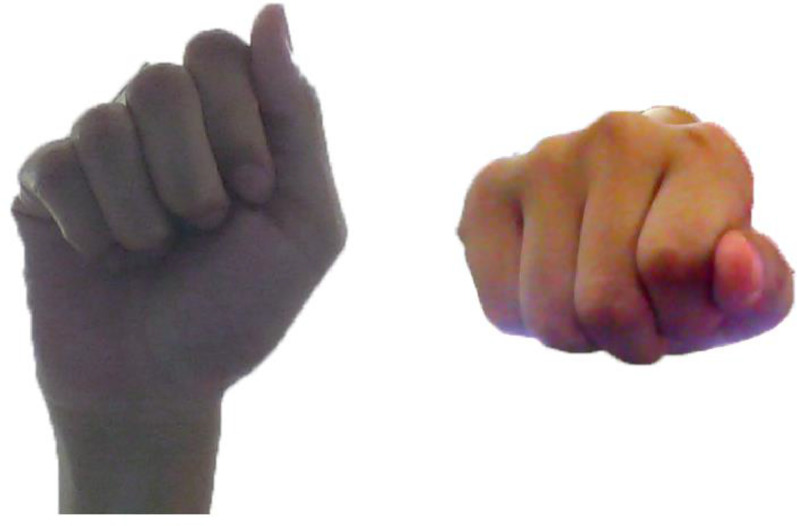
Illustration of one hand-gesture by two different views under different cameras.

There are a few hand gesture databases available to the research community. Most of the database consist of one hand gestures. Just and Marcel [5] present the first dataset for both one- and two-handed gestures. Recently Poon et al. [9,10] present a new study for bimanual (two-hands) gesture recognition to overcome the drawback of hand-hand self-occlusion. Fig. 1 illustrates an example of this phenomena in case of one hand gesture of a single gesture captured by two different cameras in front of and below the hand. Pisharady and Saerbeck [8] present a complete review methods and databases in vision-based hand gesture recognition in 26 publicly available hand gesture databases. All the reviewed databases are based on single view. By analyzing the recent published hand gesture datasets in literature (see Table 1), we see that, there is a few public hand gesture datasets dealing with multi-view cameras. The IMHG [12] is a public dataset with front view and side view for each gesture. Motivated from this idea, we propose the novel, publicly available HGM-4 for one hand gesture dataset. An illustration of 26 gestures images of HGM-4 dataset are shown in Table 2. These gestures represent the alphabet letter of Vietnamese sign language. This dataset can be applied for dealing with contactless device control application or sign language interpretation since the cameras can be disposed at any positions.

**Table 1:** Summary of the recent published hand-gesture dataset in the literature.

Dataset Name	Number of views	Number of gestures	Total images	Resolution	Publicly
FEMD [7]	1	12	1,000	640 × 480	No
Interact Play [5]	2	16	16,000	-	Yes
IMHG [12]	2	8	836	640 × 480	Yes
HGM-4	4	26	4,000	1280 × 700	Yes

**Table 2:** The 26 classes of hand gesture of HGM-4 dataset.

Gesture	Illustration	Gesture	Illustration	Gesture	Illustration
A		J		S	
B		K		T	
C		L		U	
D		M		V	
E		N		W	
F		O		X	
G		P		Y	
H		Q		Z	
I		R			

## 2 Experimental Design, Materials, and Methods

**Fig. 2:**
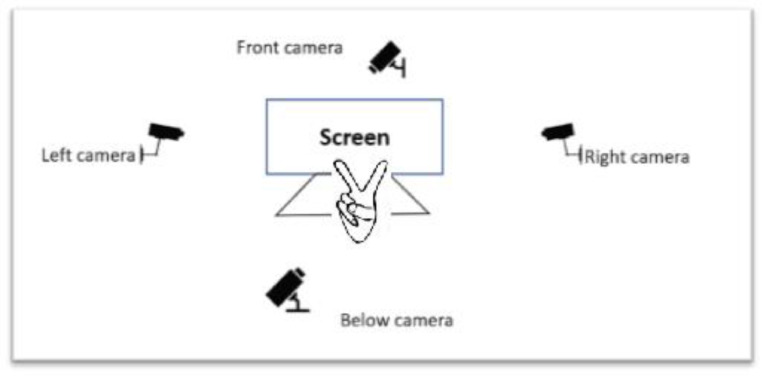
Camera setup: each hand-gesture (in front of screen and above the keyboard) is captured at the same time by four cameras.

**Fig. 3:**
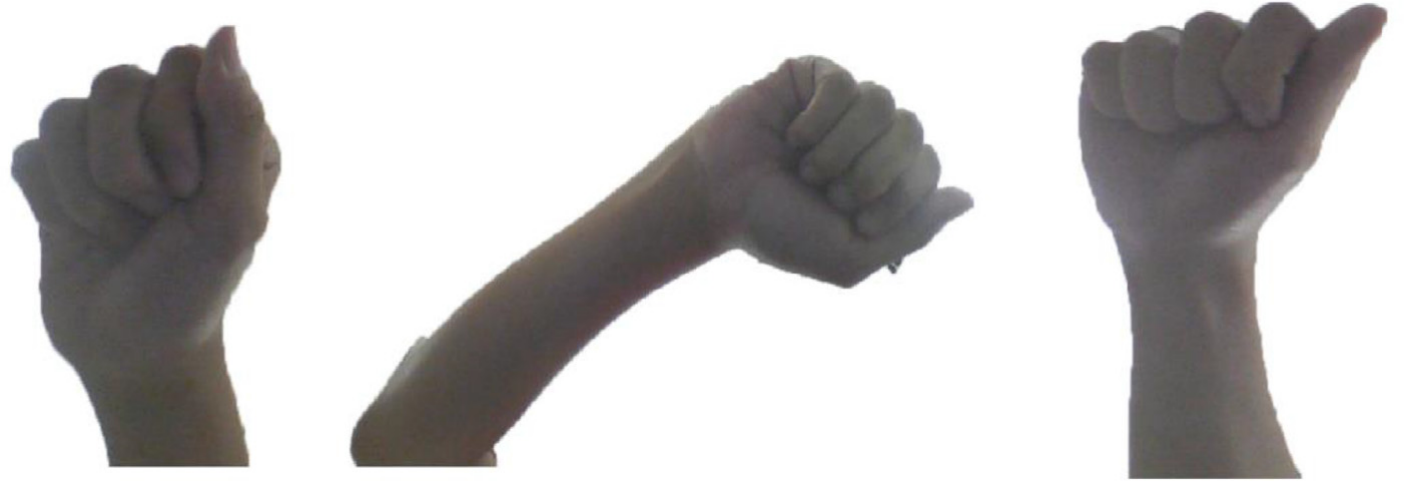
Illustration of three distinct images of the same gesture captured by below camera.

This data is available online at Mendeley Repertory. It is organized in four main folders: CAM _Left, CAM_Right, CAM_Front and CAM_Below. Each main folder contains 26 sub-folders corresponding 26 classes of hand-gestures. Each sub-folder (from A to Z) has exactly 40 colored images with 1280 × 700 pixels. Table 3 presents the properties of HGM-4 dataset. Each gesture is performed by 5 persons. Four cameras have been used to capture hand gesture at four different positions. The cameras setup of our method is illustrated in Fig. 2. We have one monitor and four fixed cameras. We have 5 volunteers and each one performs 26 hand gestures. Each person performs hand gesture in front of monitor and above the keyboard. Four images are then captured for each gesture simultaneously. The first gesture is performed at the middle of four cameras. After acquiring each picture, the volunteer moves the hand with the same gesture in order to have 8 different images at different scales. A new movement must not rotate the hand compared with the first performance. Fig. 3 illustrates three distinct images of the same gesture captured by below camera. It is worth to note that this screen is used to control and view images from four cameras. A technician will take four images after verifying quality and resolution.

**Table 3:** Properties of HGM-4 dataset.

Camera Position	Total images	Number of gestures	Number of images per gesture	Number of acting persons
CAM _Left	1,040	26	8	5
CAM_Right	1,040	26	8	5
CAM_Front	1,040	26	8	5
CAM_Below	1,040	26	8	5

**Fig. 4:**
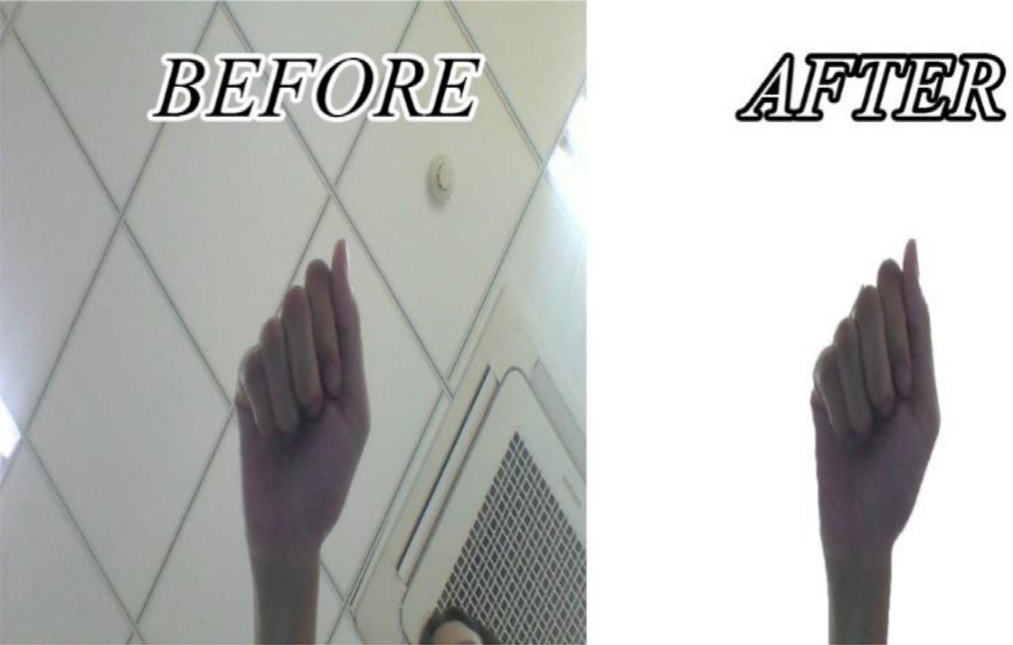
Original image and its segmentation with removed background by Otsu's method and enhanced by technical expert.

All images are segmented to remove background as illustrated in Fig. 2 by The Otsu's method [4]. This approach returns a single intensity threshold that separate pixels into two classes, foreground, and background (as illustrated in Fig. 4). The automated background removal tool is applied automatically based on selecting bimodal histogram. We use Matlab program to perform this task. However, it does not give a perfect result. In some cases, it still contains the pixels of another object or pixels of hand is removed unintentionally. Our technician verifies each image and enhance the background removal again by Photoshop program.

The standard protocol is computed by the average accuracy over 4 decomposition. In each time, all the images from one camera are used for testing set while images from the rest cameras are used for training sets. The purpose is to learn, and model a given physical images captured from different condition such as view, distance, or cameras. Three configurations are proposed, with one, two and three training sets per gesture with all possible combinations is listed in Table 4 such as:

**Table 4:** All combination possible to create training and testing set for experiment.

For one training set		For two training sets		For three training sets	
Training	Testing	Training	Testing	Training	Testing
CAM _Left	CAM _Right CAM _Front CAM _Below	CAM _Front CAM _Below	CAM _Left CAM _Right	CAM _Right CAM _Front CAM _Below	CAM _Left
CAM_Right	CAM _Left CAM _Front CAM _Below	CAM _Left CAM _Right	CAM _Front CAM _Below	CAM _Left CAM _Front CAM _Below	CAM_Right
CAM_Front	CAM _Right CAM_Left CAM _Below	CAM_ Below CAM _Left	CAM _Front CAM _Right	CAM _Right CAM_Left CAM _Below	CAM_Front
CAM_Below	CAM _Right, CAM_Left, CAM _Front	CAM _Front CAM _Right	CAM_ Below CAM _Left	CAM _Right CAM_Left CAM _Front	CAM_Below
		CAM _Left CAM _Front	CAM _Right CAM _Below		
		CAM _Right CAM _Below	CAM _Left CAM _Front		

## Uncited References:

[11,14]

## Conflict of Interest

The authors declare that they have no known competing financial interests or personal relationships that could have appeared to influence the work reported in this paper.

## Appendix Supplementary materials

Image, application 1
